# Author Correction: A CK2 and SUMO-dependent, PML NB-involved regulatory mechanism controlling BLM ubiquitination and G-quadruplex resolution

**DOI:** 10.1038/s41467-024-45551-1

**Published:** 2024-02-05

**Authors:** Shichang Liu, Erin Atkinson, Adriana Paulucci-Holthauzen, Bin Wang

**Affiliations:** 1 Department of Genetics, The University of Texas M.D. Anderson Cancer Center, Houston, TX 77030 USA; 2 Genetics and Epigenetics Program, The MD Anderson Cancer Center and UT Health Graduate School of Biomedical Sciences, Houston, TX 77030 USA

**Keywords:** DNA metabolism, Ubiquitin ligases, Sumoylation, PML bodies, Phosphorylation

Correction to: *Nature Communications* 10.1038/s41467-023-41705-9, published online 30 September 2023

In the original version of the published article, the Acknowledgements section was missing a statement about the graduate program of one author: E.A. is a Graduate Scholar in the CPRIT Training Program (RP210028).

The correct Acknowledgements paragraph is:

We thank Drs. Nathan Ellis (Memorial Sloan-Kettering Cancer Center) and Stephen Ekker (Institute of Human Genetics of University of Minnesota) for BLM and related plasmids. S.L., E.A., and B.W. were partially supported by NIH grant (CA155025, CA248088). E.A. is a Graduate Scholar in the CPRIT Training Program (RP210028). Use of the Nikon A1 microscope was made possible via a NIH shared Instrumentation Grant (grant no. 1S10OD024976-01 to the MDACC Department of Genetics).

This has now been corrected both in the HTML and the PDF versions of the article.

