# Supplementary material for: A CK2 and SUMO-dependent, PML NB-involved regulatory mechanism controlling BLM ubiquitination and G-quadruplex resolution
Source: Nat Commun. 2023 Sep 30;14:6111. doi: 10.1038/s41467-023-41705-9 (PMC10542384; doi:10.1038/s41467-023-41705-9)
Supplement: Supplementary file 1 — Supplementary Information [file 41467_2023_41705_MOESM1_ESM.pdf]

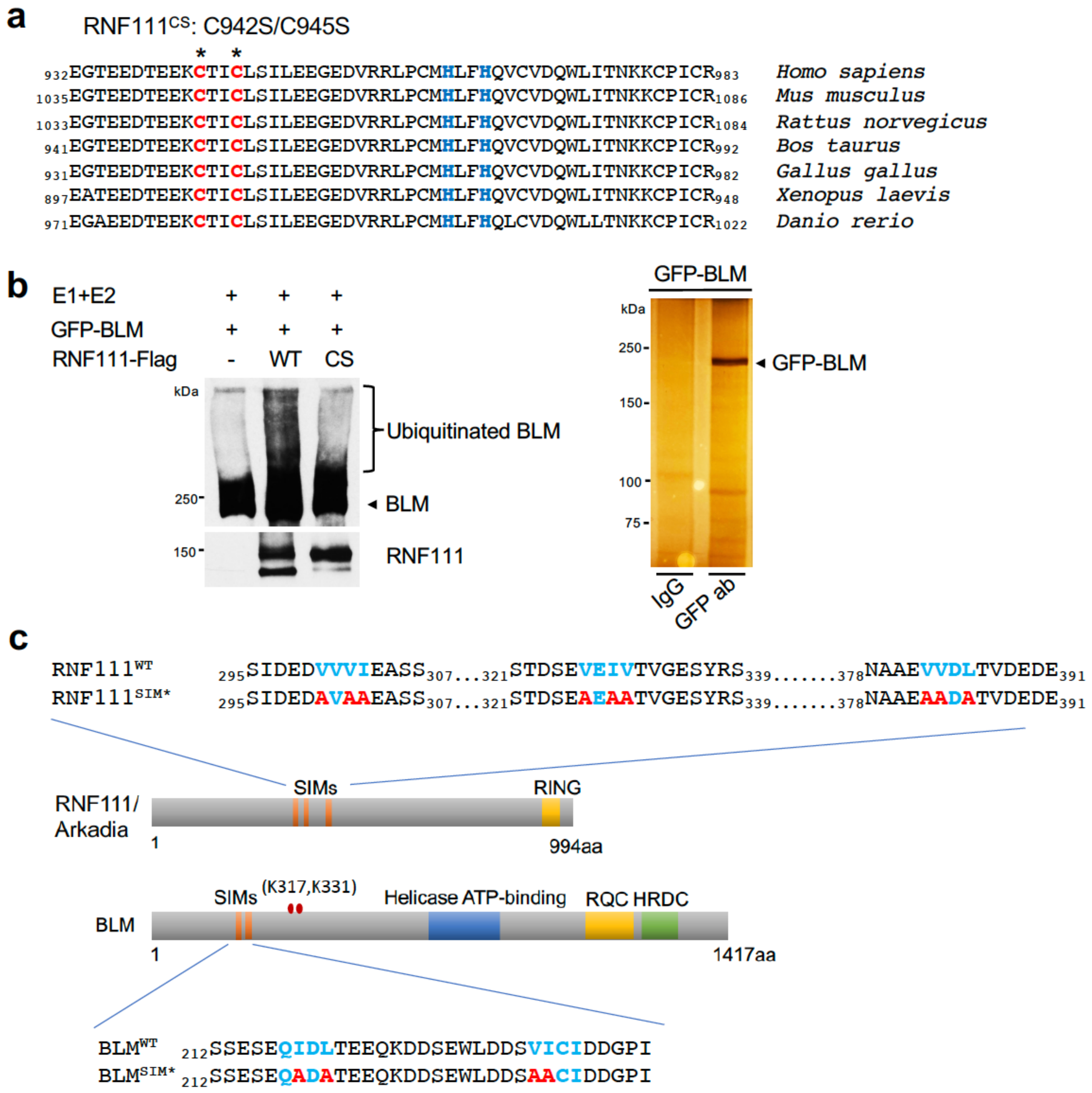

Supplementary Fig.1 (related to Fig. 1). RNF111 ubiquitinates BLM and regulates BLM levels. (**a**) Alignment of RNF111 RING domain and generation of RNF111^{CS} mutant. **(b)** RNF111 ubiquitinates purified GFP-BLM *in vitro*. In vitro ubiquitination reaction was carried out with purified GFP-BLM in the absence or presence of immunoprecipitated RNF111-Flag WT or CS mutant. Left panel, western blots were performed with antibodies to BLM or RNF111; right panel, purified GFP-BLM with silver staining. (**c**) A schematic of RNF111/Arkadia and BLM protein domains and indicated mutants. RNF111 SIMs and RING domain, as well as BLM SIMs, SUMO modification sites (K317 and K331), helicase ATP-binding, RQC (RecQ C-terminal) and HRDC (helicase-and-ribonuclease D-C-terminal) domains are shown.
Source data are provided as a Source Data file.

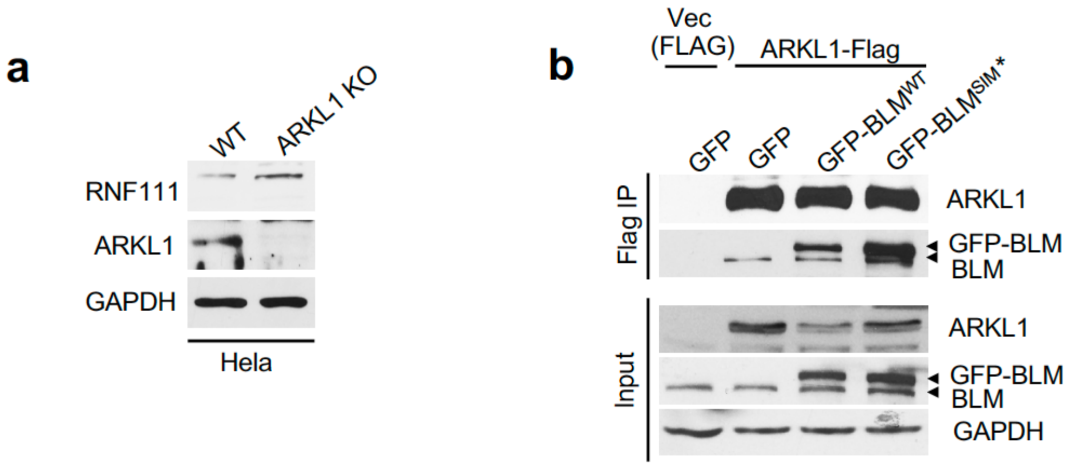

Supplementary Fig. 2 (related to Fig. 2). ARKL1 promotes BLM ubiquitination and regulates BLM protein level (**a**) Increased RNF111 levels in ARKL1 KO cells. (**b**) ARKL1-Flag interacts with BLM^{WT} or BLM^{SIM*} mutant. Flag IP was performed with lysates from 293T cells co-expressing ARKL1-Flag and GFP, GFP-BLM^{WT} or GFP-BLM^{SIM*}.
Source data are provided as a Source Data file.

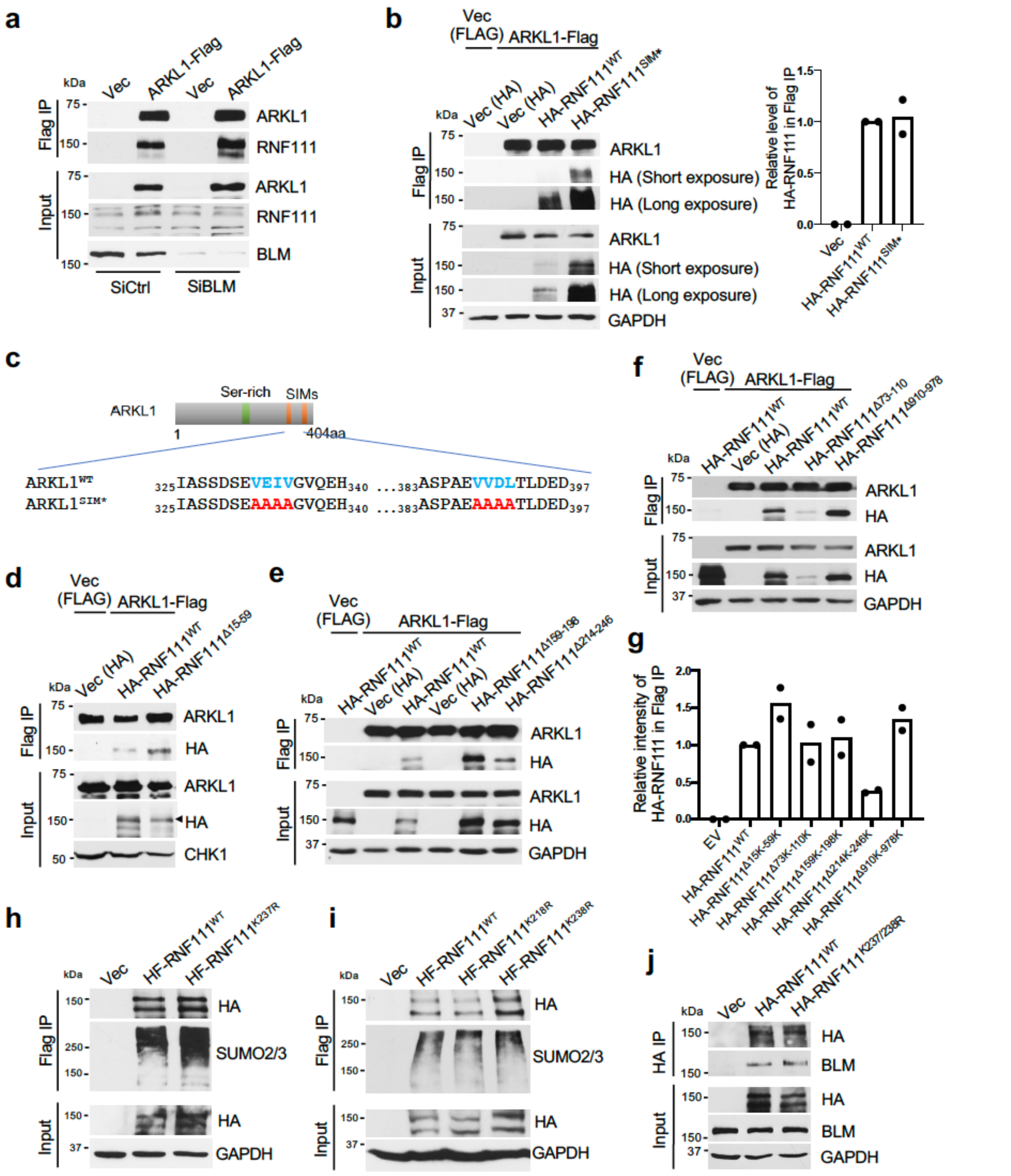

Supplementary Fig.3 (related to Fig.3). ARKL1 interacts with RNF111 in a SIM-SUMO dependent manner. (**a**) Knockdown of BLM does not affect ARKL1 and RNF111 interaction. The IPs were performed with Flag antibody using lysates of 293T cells expressing vector or ARKL1-FLAG and treated with indicated siRNAs. (**b**) The SIMs of RNF111 are not required for RNF111-ARKL1 interaction. The IPs were performed with FLAG antibody using lysates of 293T cells co-expressing ARKL1-Flag and vector, HA-RNF111^{WT} or HA-RNF111^{SIM*}. Relative level of HA-RNF111 in ARKL1-FLAG IP (normalized by input HA-RNF111 and

ARKL1-Flag immunoprecipitates) is quantified (n=2 independent experiment). **(c)** Generation of ARKL1 SIM mutant. (**d-g**) Deletion of 214-246 amino acids of RNF111 decreases RNF111 interaction with ARKL1. The IPs were performed with Flag antibody using lysates of 293T cells co-expressing ARKL1-Flag and HA-tagged RNF111 deletion mutants. Chk1 or GAPDH was used as a loading control. Relative level of HA-RNF111 in ARKL1-Flag IP (normalized by input HA-RNF111 and ARKL1-Flag immmunoprecipitates) is quantified (n=2 independent experiment). (**h**) K237R mutation alone does not affect HA-Flag-RNF111 SUMOylation. (**i**) K238R mutation alone does not affect HA-Flag-RNF111 SUMOylation. (**j**) The SUMOylation of RNF111 at K237/238 is not required for RNF111 and BLM interaction. The IPs were performed with HA antibody using lysates of 293T cells expressing vector, HA-RNF111^{WT} or HA- RNF111$^{K237/238R}$.
Source data are provided as a Source Data file.

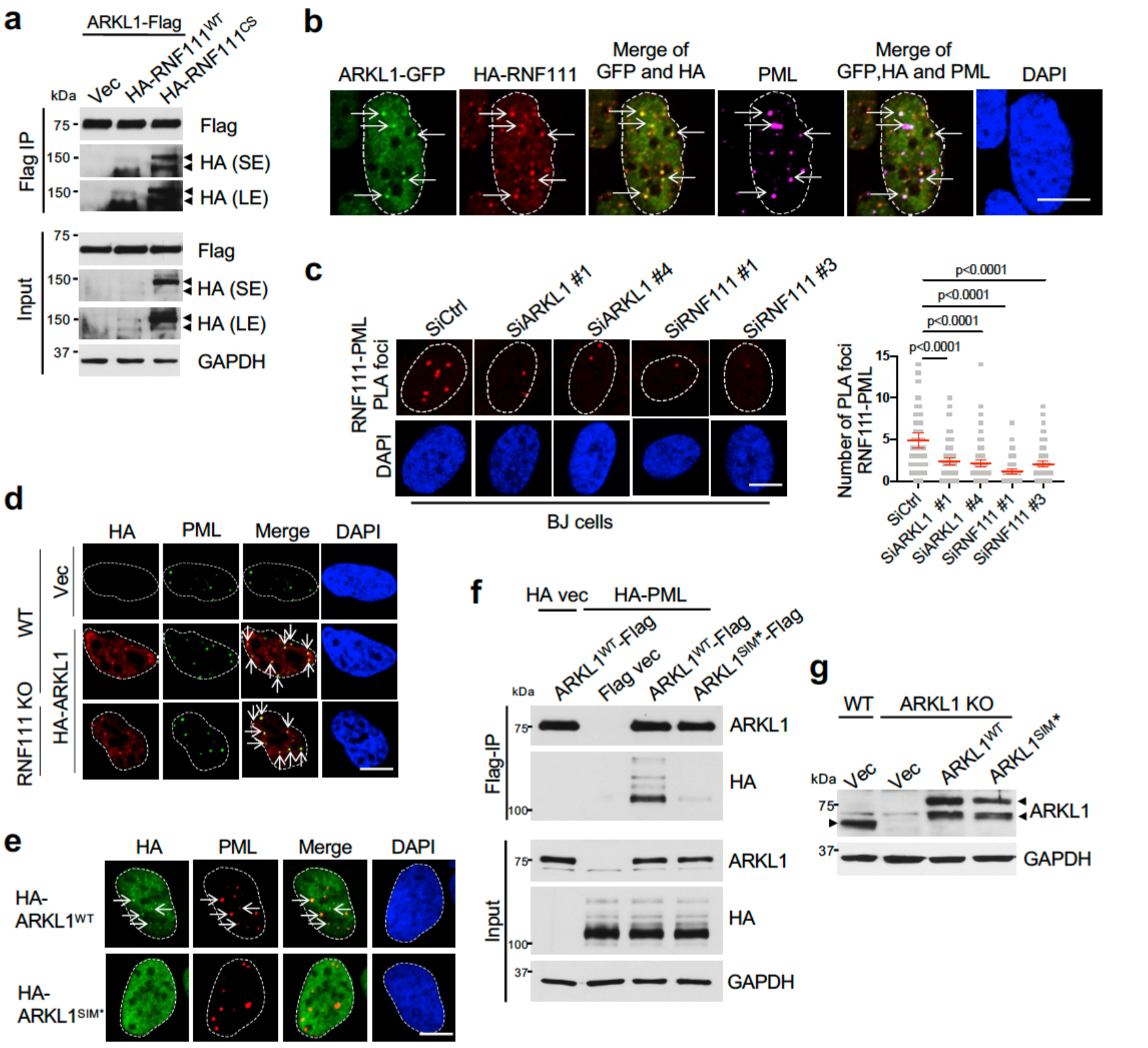

Supplementary Fig.4 (related to Fig.4). ARKL1 promotes RNF111 localization to PML NBs.
(**a**) RNF111^{CS} is intact in the interaction with ARKL1. The IPs were performed with Flag antibody using lysates from 293T cells co-expressing ARKL1-Flag and empty vector, HA-RNF111^{WT} or HA-RNF111^{CS}. **(b)** ARKL1-GFP and HA-RNF111 form foci that colocalize with PML NBs. IF was carried out with GFP, HA and PML antibodies in U2OS cells. Arrows point to the colocalized foci. (**c**) ARKL1 knockdown reduces RNF111 and PML colocalization in BJ cells. PLA was performed with RNF111 and PML antibodies. Quantification is shown with mean ± 95% CI for cells treated with indicated siRNAs, siCtrl (n=119), SiARKL1 #1 (n=122), SiARKL1 #4 (n=127), SiRNF111 #1 (n=94), SiRNF111 #3 (n=129) from two biological replicates. Statistics: one-way ANOVA with Sidak's correction. (**d**) HA-ARKL1 localization to PML NBs is independent of RNF111. IF was carried out with HA and PML antibodies in WT or RNF111 KO U2OS cells expressing empty vector or HA-ARKL1. **(e)** ARKL1^{SIM*} fails to localize to PML NBs. **(f)** ARKL1 SIM domains are required for the interaction of ARKL1-Flag with HA-PML. (**g**) ARKL1 SIM domains are required for RNF111 recruitment to PML NBs. Expression of ARKL1^{WT} and ARKL1^{SIM*} in ARKL1 KO Hela cells for Fig.4g is shown. Left arrow, endogenous ARKL1; right arrow, Flag-tagged ARKL1.
Scale bar, 10 µm for **b, c, d, e**. Source data are provided as a Source Data file.

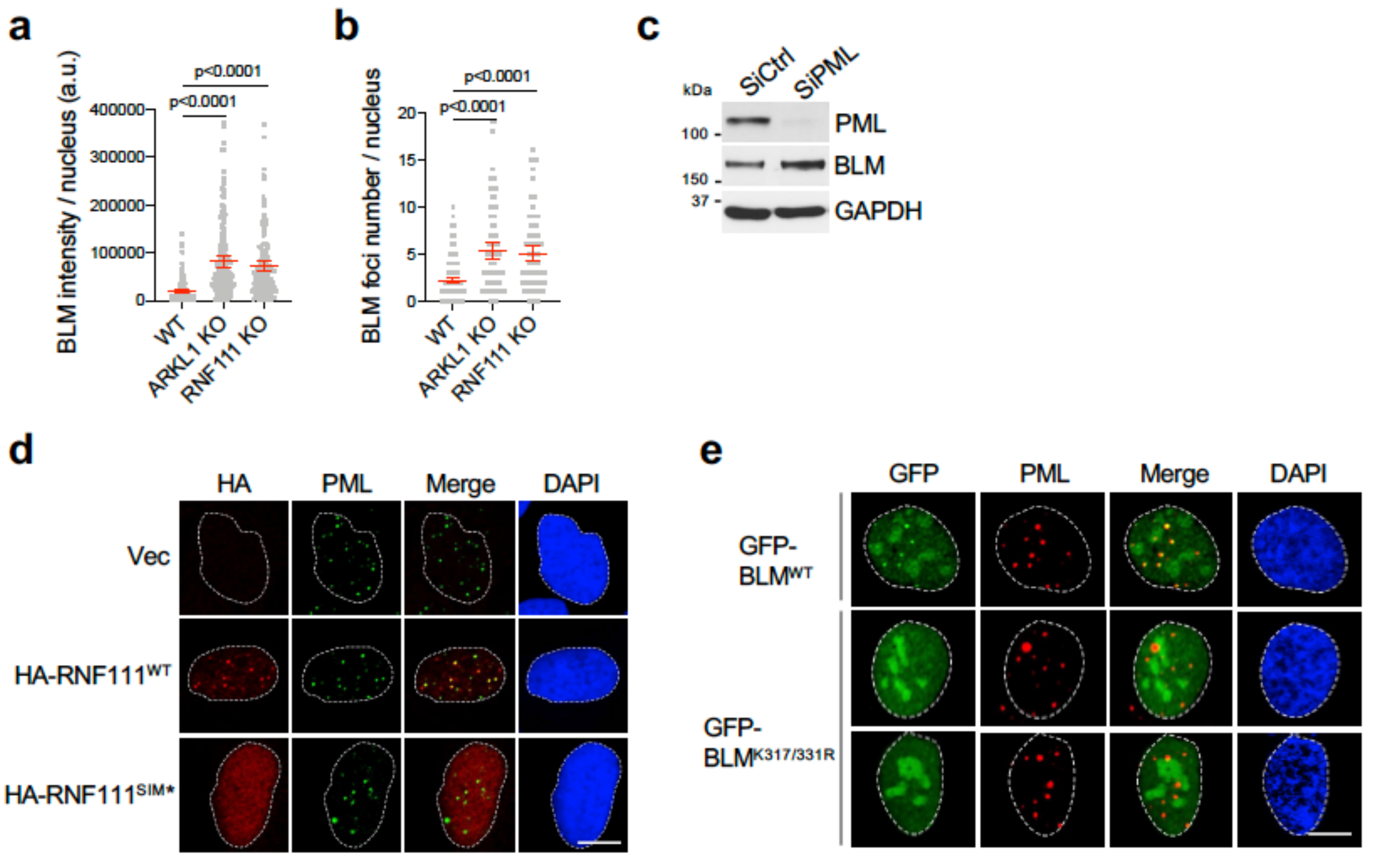

Supplementary Fig.5 (related to Fig.5). RNF111-mediated ubiquitination of BLM is associated with PML NBs. (**a**) **(b)** BLM levels and foci number are increased in ARKL1 KO and RNF111 KO U2OS cells. IF was performed with antibodies to BLM and PML. The representative images are shown in Fig. 5a. Quantification of BLM intensity and number of foci in each nucleus are shown with mean ± 95% CI for WT (n=174), ARKL1 KO (n=105), RNF111 KO (n=101) cells from two biological replicates. Statistical analysis was performed using one-way ANOVA test with Sidak's correction. **(c)** Depletion of PML leads to an elevation of BLM protein levels. **(d)** RNF111^{SIM*} mutant is deficient in localizing to PML NBs. **(e)** BLM$^{K317/331R}$ mutant is deficient in localizing to PML NBs. Scale bar, 10 µm for **d, e**.
Source data are provided as a Source Data file.

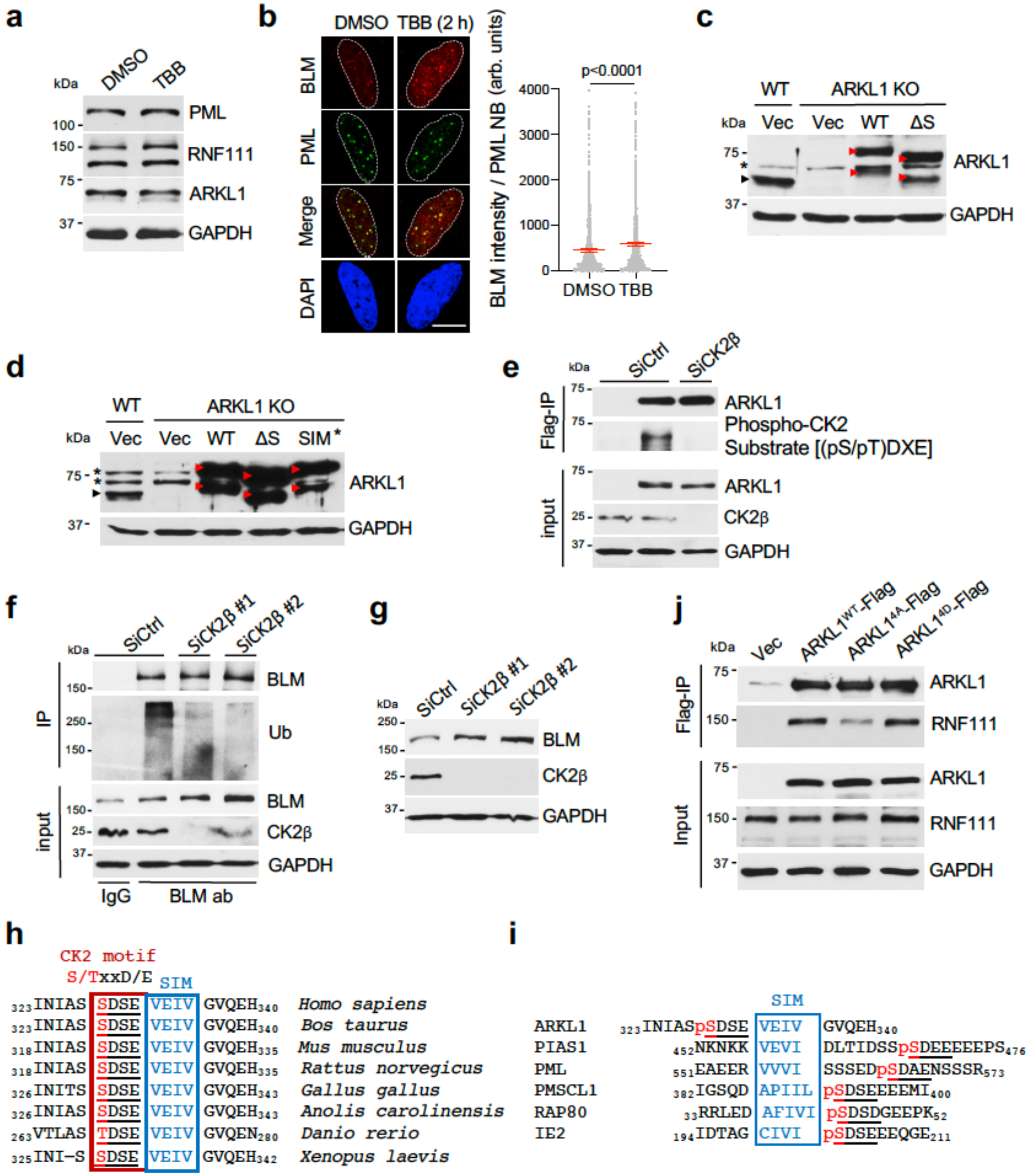

Supplementary Fig.6 (related to Fig. 6). CK2 phosphorylates ARKL1 promoting ARKL1-RNF111 interaction and BLM ubiquitination.

(**a**) TBB treatment does not reduce the protein levels of PML, RNF111 or ARKL1. U2OS cells were treated with DMSO or TBB (50µM, 4 h). GAPDH was used as a loading control. (**b**) TBB treatment (50µM, 2 h) leads to increased BLM intensity in PML NBs in U2OS cells. Quantification with arbitrary (arb.) units is shown with mean ± 95% CI for DMSO-treated (n=1351) or TBB-treated (n=1154). Statistics: two-tailed unpaired t-test. Scare bar, 10µm. (**c**) Complementation of ARKL KO U2OS cells with expression of Flag-tagged ARKL1^{WT} or ARKL1$^{\Delta S}$ (related to Fig. 6e). Black arrow, endogenous ARKL1; red arrow, ARKL1-Flag; *non-specific band.

(**d**) Western blot of WT or ARKL1 KO U2OS expressing indicated constructs used in Fig. 6f. Black arrow, endogenous ARKL1; red arrow, ARKL1-Flag; *non-specific band. **(e)** Knockdown of CK2β abolishes ARKL1 phosphorylation by CK2. Western blot was performed with phospho-CK2 substrate [(pS/pT)DXE] antibody. **(f)** Knockdown of CK2β reduces BLM ubiquitination. **(g)** Knockdown of CK2β leads to increased BLM protein levels. **(h)** CK2 phosphorylation site on ARKL1 is conserved through various species. (**i**) Alignment of CK2 phosphorylation site adjacent to a SIM present in several SIM-containing proteins. (**j**) $ARKL1^{4D}$ (S327/328/330/385D) mutant interacts with RNF111 as $ARKL1^{WT}$ does while $ARKL1^{4A}$ (S327/328/330/385A) mutant exhibits decreased interaction with RNF111. 293T cells expressing indicated constructs were used. Source data are provided as a Source Data file.

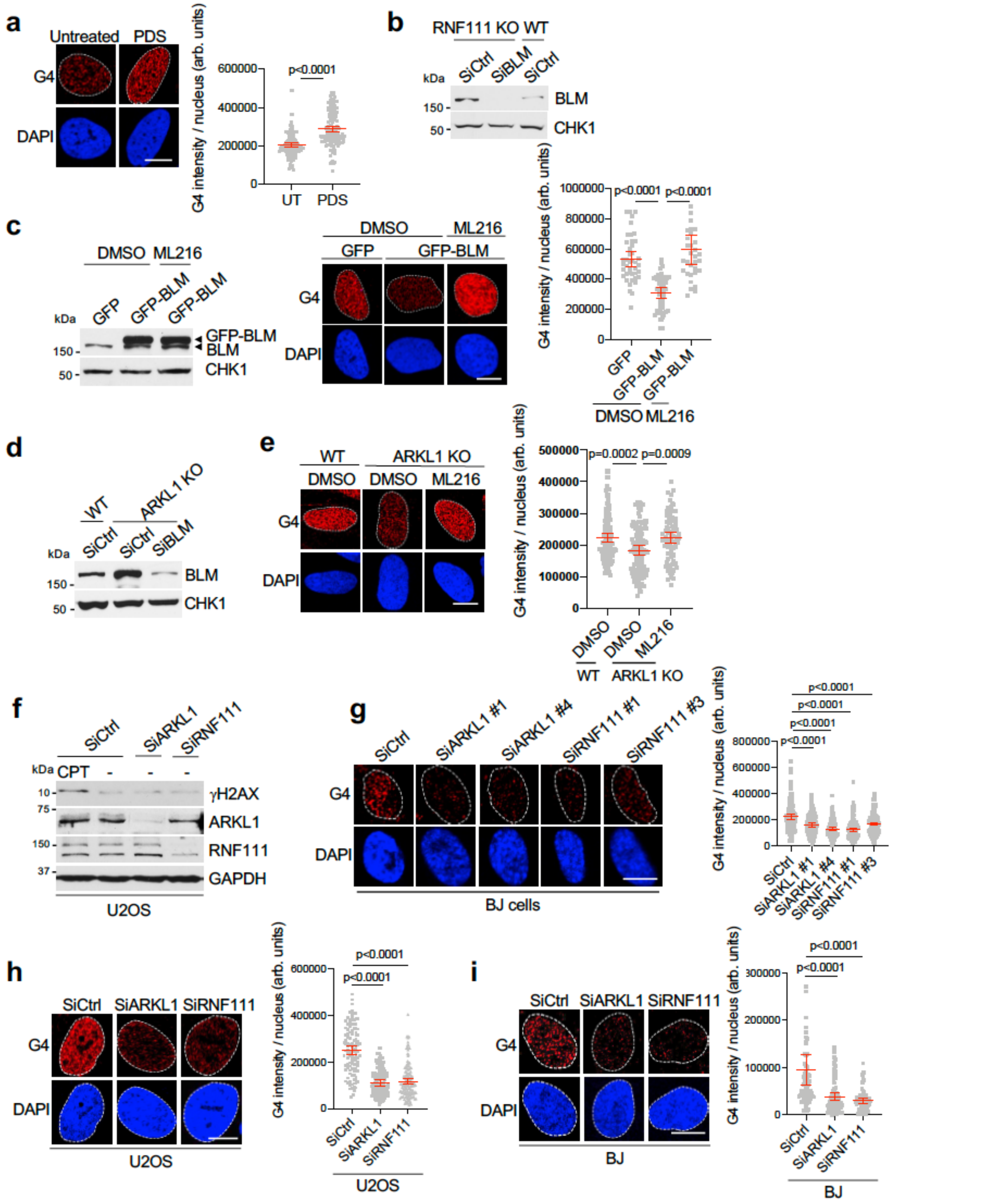

Supplementary Fig.7 (related to Fig.7). RNF111-ARKL1 regulates G-quadruplex through modulating BLM levels. (**a**) Increased G4 levels detected by BG4 antibody in U2OS cells treated with Pyridostatin (PDS) (10µM, 24h). G4 intensity was quantified with arbitrary (arb.) units for untreated (n=148) or PDS-treated (n=138) cells from two biological replicates. (**b**) Western blot of lysates from cells analyzed in Fig.7a showing

knockdown of BLM in RNF111 KO cells. CHK1 was used as a loading control. (**c**) Overexpression of GFP-BLM reduces G4 levels and the reduction is abolished by BLM helicase inhibitor ML216 (12.5μM, 48h). Expression of GFP-BLM in U2OS cells is detected by western blot with BLM antibody (left). IF was carried out with BG4 antibody (middle). Quantifications (right) are shown for cells expressing GFP treated with DMSO (n=42), GFP -BLM treated with DMSO (n=47), or ML216 (n=32). (**d**) Loss of ARKL1 reduces G4 levels in the nucleus and the reduction is abolished by BLM knockdown in U2OS cells. Western blots for Fig. 7d are shown. CHK1 was used as a loading control. (**e**) The G4 level is restored in ARKL1 KO cells treated with BLM helicase inhibitor ML216 (12.5μM, 72h). Quantifications are shown for the control U2OS WT cells treated with DMSO (n=101) or ARKL1 KO treated with DMSO (n=110) or ML216 (n=74) from two biological replicates. **(f)** Depletion of ARKL1 or RNF111 does not lead to elevated γH2AX detected by western blot. CPT (1 uM, 1 h) treatment of cells was used for a control. (**g**) G4 intensity in the nucleus is decreased in RNF111 or ARKL1 depleted BJ fibroblast cells. Quantifications of two biological replicates are shown with mean ± 95% CI for cells treated with Ctrl siRNA (n=147), SiARKL1#1 (n=118), SiARKL1#4 (n=123), SiRNF111#1 (n=112), SiRNF111#3 (n=177). **(h)** G4 intensity detected by G4 1H6 antibody in ARKL1 or RNF111-deficient U2OS cells. Quantifications of two biological replicates are shown for cells treated with Ctrl siRNA (n=112), SiARKL1 (n=94) and SiRNF111 (n=149). **(i)** G4 intensity detected by G4 1H6 antibody in ARKL1 or RNF111-deficient BJ cells. Quantifications of two biological replicates are shown for cells treated with siCtrl (n=64), SiARKL1 (n=79) and SiRNF111 (n=67).

Quantifications are shown with mean ± 95% CI for **a, c, e, g, h, i.** Statistics: two-tailed unpaired t-test for **a**, one-way ANOVA with Sidak's correction for **c, e**, **g, h, i.** Scare bar, 10μm for **a, c, e, g, h, i.** Source data are provided as a Source Data file.

Supplementary Table 1. List of siRNAs used

Name	Cat# or sequence
ON-TARGET plus Non-targeting siRNAs	Horizon, D-001810-0X
ON-TARGET plus Human C18orf25 (147339) siRNA-SMARTpool	Horizon, L-015948-02-0005
ON-TARGET plus Human RNF111 (54778) siRNA-SMARTpool	Horizon, L-007002-00-0005
siARKL1#1	GUGAAGUAAUAUUGGCUUA
siARKL1#4	AGGCAGAAACGGCGGUAGA
siRNF111#1	GUACAUACUUGCAGAUUUA
siRNF111#3	CCAAAGAUGGCAUGACUUA
siBLM	AUCAGCUAGAGGCGAUCAA
siPML	GGCUUCGACGAGUUCAA
SiCK2β #1	AGAAGAAUUCAUUGCCACGGAGCCC
SiCK2β #2	GGACAAAUUUAAUCUUACU

Supplementary Table 2. List of plasmids used

Name	Source
pcDNA3.1-ARKL1^{WT}-3xFLAG	This paper
pcDNA3.1-ARKL1^{SIM*}-3xFLAG	This paper
pcDNA3.1-ARKL1$^{\Delta S}$-3xFLAG	This paper
pcDNA3.1-ARKL1^{S328A}-3xFLAG	This paper
pcDNA3.1-ARKL1$^{S327/328/330/385A}$-3xFLAG	This paper
pcDNA3.1-ARKL1$^{S327/328/330/385D}$-3xFLAG	This paper
MSCV-3HA-ARKL1^{WT}	This paper
MSCV-3HA-ARKL1^{SIM*}	This paper
MSCV-GFP-ARKL1^{WT}	This paper
MSCV-HA-Flag-ARKL1^{WT}	This paper
pAID5.3-RNF111^{WT}-miniAID	This paper
pAID5.3-RNF111^{SIM*-}miniAID	This paper
pAID5.3-RNF111^{CS}-miniAID	This paper
pcDNA3.1-RNF111^{WT}-3xFLAG	This paper
pcDNA3.1-RNF111^{SIM*}-3xFLAG	This paper
pcDNA3.1-RNF111^{CS}-3xFLAG	This paper
MSCV-HA-Flag-RNF111^{WT}	This paper
MSCV-HA-Flag-RNF111$^{K218/237R}$	This paper
MSCV-HA-Flag-RNF111$^{K237/238R}$	This paper
MSCV-3HA-RNF111^{WT}	This paper
MSCV-3HA-RNF111$^{\Delta 15-59}$	This paper
MSCV-3HA-RNF111$^{\Delta 73-110}$	This paper
MSCV-3HA-RNF111$^{\Delta 159-198}$	This paper
MSCV-3HA-RNF111$^{\Delta 214-246}$	This paper
MSCV-3HA-RNF111$^{\Delta 910-978}$	This paper
MSCV-3HA-RNF111^{SIM*}	This paper
GFP-BLM^{SIM*}	This paper
MSCV-GFP-PML (PML isoform II)	This paper
MSCV-mcherry-PML (isoform II)	This paper
pcDNA3.1-ARKL1^{WT}-3xFLAG	This paper
GFP-BLM	A gift from Nathan Ellis (Addgene plasmid #80070)
pAID5.3-C	A gift from Masato Kanemaki (Addgene plasmid #145813)
pCMV-Tol2	A gift from Stephen Ekker (Addgene plasmid#31823)

Supplementary Table 3. List of primers used

Name	Sequence
BLM SIM1 fw	GTCATCCTTCTGTTCCTCAGTCGCATCTGCTTGCTCGCTTTCAGAGGAGGGTG
BLM SIM1 rev	CACCCTCCTCTGAAAGCGAGCAAGCAGATGCGACTGAGGAACAGAAGGATGAC
BLM SIM2 fw	GGGGCCATCATCGATGCAAGCCGCATCGCTGCTTAACCATTC
BLM SIM2 rev	GAATGGTTAAGCAGCGATGCGGCTTGCATCGATGATGGCCCC
ARKL1 SIM1 fw	CCTTGCATGTTCCTGAACTCCCGCAGCCTCTGCTTCACTATCTGAAGACGCAAT
ARKL1 SIM1 rev	ATTGCGTCTTCAGATAGTGAAGCAGAGGCTGCGGGAGTTCAGGAACATGCAAGG
ARKL1 SIM2 fw	TGGGCTTCACCAGCAGAAGCTGCTGACGCTACCTTGGATGAGGATAGC
ARKL1 SIM2 rev	GCTATCCTCATCCAAGGTAGCGTCAGCAGCTTCTGCTGGTGAAGCCCA
ARKL1 S385A fw	CAACTTCTGCTGGTGCAGCCCAAGTCTGCTG
ARKL1 S385A rev	CAGCAGACTTGGGCTGCACCAGCAGAAGTTG
ARKL1 S327/328/330A fw	ACTCAATGAAGAAATTAACATTGCGGCTGCAGATGCTGAAGTAGAGATTGTGGGAGTTCAG
ARKL1 S327/328/330A rev	CTGAACTCCCACAATCTCTACTTCAGCATCTGCAGCCGCAATGTTAATTTCTTCATTGAGT
ARKL1 S385D fw	GTCAACAACTTCTGCTGGATCAGCCCAAGTCTGCTGGGGAG
ARKL1 S385D rev	CTCCCCAGCAGACTTGGGCTGATCCAGCAGAAGTTGTTGAC
ARKL1 S327/328/330D fw	AATGCGCCACTCAATGAAGAAATTAACATTGCGGATGATGATGATGAAGTAGAGATTGTGGGAGTTCAGGAACATGC
ARKL1 S327/328/330D rev	GCATGTTCCTGAACTCCCACAATCTCTACTTCATCATCATCATCCGCAATGTTAATTTCTTCATTGAGTGGCGCATT
ARKL1 S328A fw	CACAATCTCTACTTCACTATCTGCAGACGCAATGTTAATTTCTTCAT
ARKL1 S328A rev	ATGAAGAAATTAACATTGCGTCTGCAGATAGTGAAGTAGAGATTGTG
ARKL1 ΔS fw	AAGGAAGAAATATAACCTGCTGGATGATGATGAAGAGGTTTCAG
ARKL1 ΔS rev	CTGAAACCTCTTCATCATCATCCAGCAGGTTATATTTCTTCCTT
RNF111 SIM1 fw	TGGGGAGTGGAGGAAGCTTCTGCCGCCACAGCATCTTCATCAATACTTCCTG

RNF111 SIM1 rev	CAGGAAGTATTGATGAAGATGCTGTGGCGGCAGAAGCTTCCTCCACTCCCCA
RNF111 SIM2 fw	CCGATAGCTTTCTCCAACTGTTGCAGCCTCCGCTTCACTGTCAGTTGAGGTAAC
RNF111 SIM2 rev	GTTACCTCAACTGACAGTGAAGCGGAGGCTGCAACAGTTGGAGAAAGCTATCGG
RNF111 SIM3 fw	GTTCATCTTCATCAACGGTAGCGTCCGCAGCTTCTGCTGCATTCTGCCTC
RNF111 SIM3 rev	GAGGCAGAATGCAGCAGAAGCTGCGGACGCTACCGTTGATGAAGATGAAC
RNF111 C943/946S fw	CTTCCTCTAAAATAGACAAACTGATAGTACTTTTTTCCTCTGTGTCTTCCTCA
RNF111 C943/946S rev	TGAGGAAGACACAGAGGAAAAAAGTACTATCAGTTTGTCTATTTTAGAGGAAG
RNF111 Δ15-59 fw	CCTGAATATAACGAGCTCTACACCTTAGTGGGGAATGAATTC
RNF111 Δ1-59 rev	GAATTCATTCCCCACTAAGGTGTAGAGCTCGTTATATTCAGG
RNF111 Δ73-110 fw	TCCTAATATTCCCTGGTTTTCTTGAGAATCATCACACAGGTG
RNF111 Δ73-110 rev	CACCTGTGTGATGATTCTCAAGAAAACCAGGGAATATTAGGA
RNF111 Δ159-198 fw	CATGTAAACAGGGTCTATCCTCATCTGAAGTCACAGTATCAG
RNF111 Δ159-198 rev	CTGATACTGTGACTTCAGATGAGGATAGACCCTGTTTACATG
RNF111 Δ214-246 fw	GGAGACTTCCATGCAGATATGCCTTGCTACCTAG
RNF111 Δ214-246 rev	CTAGGTAGCAAGGCATATCTGCATGGAAGTCTCC
RNF111 Δ910-978 fw	GGACAATTGAAAGATGTACATATCCACATTGCCCCATATGCAGA
RNF111 Δ910-978 rev	TCTGCATATGGGGCAATGTGGATATGTACATCTTTCAATTGTCC

Supplementary Table 4. List of antibodies used

Name	Cat #	Dilution
BLM Antibody (C-1)	Santa Cruz, sc-376237	1:200 for IF or PLA<br>1:200 for WB
BLM Antibody (C-18)	Santa Cruz, sc-7790	1:200 for IF or PLA
HA-Tag Antibody (C29F4)	Cell Signaling, 3724S	1:800 for IF or PLA<br>1:2000 for WB
HA-Tag (6E2)	Cell Signaling, 2367S	1:800 for IF or PLA<br>1:2000 for WB
PML Antibody (PG-M3)	Santa Cruz, sc-966	1:200 for IF or PLA
GFP Antibody (1A5)	Santa Cruz, sc-101536	1:200 for IF
DNA G-quadruplex structures Antibody (BG4)	Sigma-Aldrich, MABE917	1:200 for IF
DNA G-quadruplex (G4) Antibody (1H6) ZooMAb® Mouse Monoclonal	Sigma-Aldrich, ZMS1070	1:600 for IF
FLAG antibody	Millipore, F7425	1:600 for IF<br>1:1000 for WB
ARKL1 antibody	Home-made	1:1000 for IF or PLA<br>1:10000 for WB
RNF111 antibody	Sigma-Aldrich, HPA038576	1:600 for IF or PLA<br>1:2000 for WB
BLM Antibody	Bethyl Laboratories, A300-110A	1:2000 for WB
PML Antibody (E-11)	Santa Cruz, sc-377390	1:100 for WB
RNF111 antibody (M05)	Abnova, H00054778-M05	1:500 for WB
GAPDH Antibody (GA1R)	Thermo Fisher Scientific, MA5-15738	1:10000 for WB
Chk1 Antibody (G-4)	Santa Cruz, sc-8408	1:200 for WB
SUMO-2/3 antibody (18H8)	Cell Signaling, 4971S	1:2000 for WB
Ubiquitin Antibody (P4D1)	Santa Cruz, sc-8017	1:200 for WB
Phospho-CK2 Substrate [(pS/pT)DXE] antibody	Cell Signaling, 8738	1:1000 for WB
Casein kinase IIβ (6D5) antibody	Santa Cruz, sc-12739	1:200 for WB
γH2AX antibody	Millipore, 05-636	1:1000 for WB
Duolink™ In Situ PLA Probe Anti-Rabbit MINUS	Sigma-Aldrich, DUO92005	1:5 for PLA
Duolink™ In Situ PLA Probe Anti-Mouse PLUS	Sigma-Aldrich, DUO92001	1:5 for PLA
Goat anti-Mouse IgG (H+L) Highly Cross-Adsorbed Secondary Antibody, Alexa Fluor 647	Thermo Fisher Scientific, A-21236	1:1000 for IF
Goat anti-Rabbit IgG (H+L) Highly Cross-Adsorbed Secondary Antibody, Alexa Fluor 647	Thermo Fisher Scientific, A-21245	1:1000 for IF

Donkey anti-Mouse IgG (H+L) Highly Cross-Adsorbed Secondary Antibody, Alexa Fluor 488	Thermo Fisher Scientific, A-21202	1:1000 for IF
Goat anti-Rabbit IgG (H+L) Cross-Adsorbed Secondary Antibody, Alexa Fluor 488	Thermo Fisher Scientific, A-11008	1:1000 for IF
Goat anti-Rat IgG (H+L) Cross-Adsorbed Secondary Antibody, Alexa Fluor 488	Thermo Fisher Scientific, A-11006	1:1000 for IF
Donkey anti-Rabbit IgG (H+L) Highly Cross-Adsorbed Secondary Antibody, Alexa Fluor 555	Thermo Fisher Scientific, A-31572	1:1000 for IF
Donkey anti-Goat IgG (H+L) Cross-Adsorbed Secondary Antibody, Alexa Fluor 555	Thermo Fisher Scientific, A-21432	1:1000 for IF
Donkey anti-Mouse IgG (H+L) Highly Cross-Adsorbed Secondary Antibody, Alexa Fluor 555	Thermo Fisher Scientific, A-31570	1:1000 for IF
