# Supplementary material for: A CK2 and SUMO-dependent, PML NB-involved regulatory mechanism controlling BLM ubiquitination and G-quadruplex resolution
Source: Nat Commun. 2023 Sep 30;14:6111. doi: 10.1038/s41467-023-41705-9 (PMC10542384; doi:10.1038/s41467-023-41705-9)
Supplement: Supplementary file 3 — Reporting Summary [file 41467_2023_41705_MOESM3_ESM.pdf]

nature portfolio

Corresponding author(s): Bin Wang, NCOMMS-23-03846A

Last updated by author(s): Aug 30, 2023

Reporting Summary

Nature Portfolio wishes to improve the reproducibility of the work that we publish. This form provides structure for consistency and transparency in reporting. For further information on Nature Portfolio policies, see our Editorial Policies and the Editorial Policy Checklist.

Statistics

For all statistical analyses, confirm that the following items are present in the figure legend, table legend, main text, or Methods section.

n/a	Confirmed	
☐	☒	The exact sample size (*n*) for each experimental group/condition, given as a discrete number and unit of measurement
☐	☒	A statement on whether measurements were taken from distinct samples or whether the same sample was measured repeatedly
☐	☒	The statistical test(s) used AND whether they are one- or two-sided<br>*Only common tests should be described solely by name; describe more complex techniques in the Methods section.*
☒	☐	A description of all covariates tested
☒	☐	A description of any assumptions or corrections, such as tests of normality and adjustment for multiple comparisons
☐	☒	A full description of the statistical parameters including central tendency (e.g. means) or other basic estimates (e.g. regression coefficient) AND variation (e.g. standard deviation) or associated estimates of uncertainty (e.g. confidence intervals)
☐	☒	For null hypothesis testing, the test statistic (e.g. *F*, *t*, *r*) with confidence intervals, effect sizes, degrees of freedom and *P* value noted<br>*Give P values as exact values whenever suitable.*
☒	☐	For Bayesian analysis, information on the choice of priors and Markov chain Monte Carlo settings
☒	☐	For hierarchical and complex designs, identification of the appropriate level for tests and full reporting of outcomes
☒	☐	Estimates of effect sizes (e.g. Cohen's *d*, Pearson's *r*), indicating how they were calculated

Our web collection on statistics for biologists contains articles on many of the points above.

Software and code

Policy information about availability of computer code

Data collection	Images of immunofluorescence, proximity ligation assay and fluorescence recovery after photobleaching were collected by NIS-Elements AR software (AR 5.21.03 64bit) of Nikon A1-Confocal
Data analysis	Image J 1.52q was used to quantify the data of immunofluorescence and proximity ligation assay. GraphPad Prism 9 (for macOS, version 9.0.0 (86)) and Microsoft Excel (Version 16.16.27) were used for statistical analysis. Photoshop CC 2018 was used to process images. Microsoft PowerPoint (Version 16.16.27) was used to draw figures.

For manuscripts utilizing custom algorithms or software that are central to the research but not yet described in published literature, software must be made available to editors and reviewers. We strongly encourage code deposition in a community repository (e.g. GitHub). See the Nature Portfolio guidelines for submitting code & software for further information.

Data

Policy information about availability of data

All manuscripts must include a data availability statement. This statement should provide the following information, where applicable:

- Accession codes, unique identifiers, or web links for publicly available datasets
- A description of any restrictions on data availability
- For clinical datasets or third party data, please ensure that the statement adheres to our policy

Source data are provided with this study. All data supporting the findings of this study are available within the paper and its supplementary information. All other data supporting the findings of this study are available from the corresponding author on reasonable request.

Human research participants

Policy information about studies involving human research participants and Sex and Gender in Research.

Reporting on sex and gender	n/a
Population characteristics	n/a
Recruitment	n/a
Ethics oversight	n/a

Note that full information on the approval of the study protocol must also be provided in the manuscript.

Field-specific reporting

Please select the one below that is the best fit for your research. If you are not sure, read the appropriate sections before making your selection.

☒ Life sciences ☐ Behavioural & social sciences ☐ Ecological, evolutionary & environmental sciences

For a reference copy of the document with all sections, see nature.com/documents/nr-reporting-summary-flat.pdf

Life sciences study design

All studies must disclose on these points even when the disclosure is negative.

Sample size	Sample size was not pre-determined using statistics tools. Sample sizes was chosen to be adequate based on the magnitude and consistency of measurable differences between groups. Minimal size of analyzed biological samples was two. For IF staining and microscopy-based experiments, more than 100 cells per experiment were counted for statistical analyses except those indicated in the figure legends. Statistical analysis (as described in respective figure legends) was used to calculate statistical significance of obtained results. The individual p-values are indicated in figures.
Data exclusions	No data were excluded from the analyses.
Replication	All experiments were repeated at least two or three times. Similar results were obtained from each independent experiments
Randomization	There was no randomization of samples because randomization does not apply to cell-based experiments in which samples are dependent upon specific genetic cell lines or knockdown conditions. For microscopy- based experiments, we randomly selected fields for image capture by using only the DAPI channel.
Blinding	Blinding was applied for quantification of microscopy- based images (without knowing the samples names but only the ID number of images). Blinding was not applied for western blot based experiments or other experiments as all data are obtained and presented in an unbiased way.

Reporting for specific materials, systems and methods

We require information from authors about some types of materials, experimental systems and methods used in many studies. Here, indicate whether each material, system or method listed is relevant to your study. If you are not sure if a list item applies to your research, read the appropriate section before selecting a response.

Materials & experimental systems

n/a	Involved in the study
☐	☒ Antibodies
☐	☒ Eukaryotic cell lines
☒	☐ Palaeontology and archaeology
☒	☐ Animals and other organisms
☒	☐ Clinical data
☒	☐ Dual use research of concern

Methods

n/a	Involved in the study
☒	☐ ChIP-seq
☒	☐ Flow cytometry
☒	☐ MRI-based neuroimaging

Antibodies

Antibodies used

Primary and secondary antibodies used for immunofluoscence staining or proximity ligation assay:
BLM Antibody (C-1) (Santa Cruz, sc-376237, 1:200)
BLM Antibody (C-18) (Santa Cruz, sc-7790, 1:200)
HA-Tag Antibody (C29F4) (Cell Signaling, 3724S, 1:800)
HA-Tag (6E2) (Cell Signaling, 2367S, 1:800)
PML Antibody (PG-M3) (Santa Cruz, sc-966, 1:200)
GFP Antibody (1A5) (Santa Cruz, sc-101536, 1:200)
DNA G-quadruplex structures Antibody (BG4) (Sigma-Aldrich, MABE917, 1:200)
DNA G-quadruplex (G4) Antibody (1H6) ZooMAb® Mouse Monoclonal (Sigma-Aldrich, ZMS1070, 1:600)
FLAG antibody (Millipore, F7425, 1:600)
ARKL1 antibody (home-made, 1:1000)
RNF111 antibody (Sigma-Aldrich, HPA038576, 1:600)

Goat anti-Mouse IgG (H+L) Highly Cross-Adsorbed Secondary Antibody, Alexa Fluor 647 (Thermo Fisher Scientific, A-21236, 1:1000)
Goat anti-Rabbit IgG (H+L) Highly Cross-Adsorbed Secondary Antibody, Alexa Fluor 647 (Thermo Fisher Scientific, A-21245, 1:1000)
Donkey anti-Mouse IgG (H+L) Highly Cross-Adsorbed Secondary Antibody, Alexa Fluor 488 (Thermo Fisher Scientific, A-21202, 1:1000)
Goat anti-Rabbit IgG (H+L) Cross-Adsorbed Secondary Antibody, Alexa Fluor 488 (Thermo Fisher Scientific, A-11008, 1:1000)
Goat anti-Rat IgG (H+L) Cross-Adsorbed Secondary Antibody, Alexa Fluor 488 (Thermo Fisher Scientific, A-11006, 1:1000)
Donkey anti-Rabbit IgG (H+L) Highly Cross-Adsorbed Secondary Antibody, Alexa Fluor 555 (Thermo Fisher Scientific, A-31572, 1:1000)
Donkey anti-Goat IgG (H+L) Cross-Adsorbed Secondary Antibody, Alexa Fluor 555 (Thermo Fisher Scientific, A-21432, 1:1000)
Donkey anti-Mouse IgG (H+L) Highly Cross-Adsorbed Secondary Antibody, Alexa Fluor 555 (Thermo Fisher Scientific, A-31570, 1:1000)

Primary antibodies used for western blot:
BLM Antibody (Bethyl Laboratories, A300-110A, 1:2000)
BLM Antibody (C-1) (Santa Cruz, sc-376237, 1:200)
HA-Tag Antibody (C29F4) (Cell Signaling, 3724S, 1:2000)
HA-Tag (6E2) (Cell Signaling, 2367S, 1:2000)
PML Antibody ((E-11) (Santa Cruz, sc-377390, 1:200)
RNF111 antibody (Sigma-Aldrich, HPA038576, 1:2000)
RNF111 antibody (M05) (Abnova, H00054778-M05, 1:500)
GAPDH Antibody (GA1R) (Thermo Fisher Scientific, MA5-15738, 1:10000)
ARKL1 antibody (home-made, 1:10000)
Chk1 Antibody (G-4) (Santa Cruz, sc-8408, 1:200)
SUMO-2/3 antibody (18H8) (Cell Signaling, 4971S, 1:2000)
Ubiquitin Antibody (P4D1) (Santa Cruz, sc-8017, 1:200)
Phospho-CK2 Substrate [(pS/pT)DXE] antibody (Cell Signaling, 8738, 1:1000)
FLAG antibody (Millipore, F7425, 1:1000)
Casein kinase IIβ (6D5) antibody (Santa Cruz, sc-12739, 1:200)
γH2AX antibody (Millipore, 05-636, 1:1000)

Validation

Antibodies used for immunofluorescence staining and proximity ligation assay were validated by the manufacturer or used in reference, and respective IF images can be found:
BLM Antibody (C-1) (Fig 3b, 5b, 6d, 6f, S6b)
BLM Antibody (C-18) (Fig 5a)
HA-Tag Antibody (C29F4) (Fig 4e, 4h, 6a, 6b, S4b, S4d)
HA-Tag (6E2) (Fig 3d, 3j)
PML Antibody (PG-M3) (Fig 4e, 4f, 4g, 4h, 5a, 6a, 6b, 6d, 6e, 6f, S4b, S4c, S4d, S6b)
GFP Antibody (1A5) (Fig 4d, 5b, S4b)
DNA G-quadruplex structures Antibody (BG4) (Fig 7a, 7b, 7c, 7d, 7e, S7a, S7c, S7e, S7f)
FLAG antibody (Fig 7a, 7b, 7c, 7d, 7e, S7a, S7c, S7e, S7f)
ARKL1 antibody (Fig 3b, F3j,4d,)
RNF111 antibody (Fig 3d, 4d, F4f, 4g, 5b, 6e, S4c)

Primary antibodies used for western blot were validated by the manufacturer.
BLM Antibody (Fig 1a, 1b, 1c, 1d, 1e, 1g, 1h, 2b, 2c, 2d, 2g, 3a, 3c, 5c, 5d, 5f, 5g, 5h, 5i, 6c, S1b, S2b, S3a, S3j, S5c, S6f, S6g, S7b, S7c, S7d)
HA-Tag Antibody (C29F4) (Fig 1g, 2g, 3a, 3f, 3h, 3i, 5e, 6h, 6i, S3b, S3d, S3e, S3f, S3h, S3i, S3j, S4a, S4f)
PML Antibody ((E-11) (Fig S5c, S6a)

RNF111 antibody (HPA038576) (Fig 1a, 1b, 1c, 1d, 1e, 1h, 2e, 2f, 2g, 3a, 3c, 5c, 5d, 5i, 6j, 6k, S2a, S3a, S6a, S6j, S7i)
RNF111 antibody (M05) (Fig 2f)
GAPDH Antibody (GA1R) (Fig 1a, 1b, 1c, 1e, 1f, 1g, 2b, 2c, 2d, 2e, 2g, 3f, 3h, 5e, 5f, 5h, 6g, 6h, S2a, S2b, S3b, S3d, S3e, S3f, S3h, S3i, S3j, S4a, S4g, S5c, S6a, S6c, S6d, S6f, S6g, S6j, S7i)
ARKL1 antibody (Fig 2b, 2c, 2d, 2f, 3c, 3i, 5g, 5h, 6g, 6j, 6k, S2a, S2b, S3a, S3b, S3d, S3e, S3f, S4f, S4g, S6a, S6c, S6d, S6e, S6j, S7i)
Chk1 Antibody (G-4) (Fig 3i, 5c, 5d, 5i, 6c, 6i, S7b, S7c, S7d)
SUMO-2/3 antibody (18H8) (Fig 3f, 3h, 5f, S3h, S3i)
Ubiquitin Antibody (P4D1) (Fig 1d, 1e, 1f, 2d, 2f, 5c, 5e, 5f, 5g, 5i, 6c, S6f)
Phospho-CK2 Substrate [(pS/pT)DXE] antibody (Fig 6g, 6h, 6i, S6e)
FLAG antibody (Fig 1f, 3c, S4a)
casein kinase IIβ (6D5) antibody (Fig S6e, S6f, S6g)
γH2AX antibody (S7i)

Eukaryotic cell lines

Policy information about cell lines and Sex and Gender in Research

Cell line source(s)	U2OS (HBT-96), Hela CCL-2), BJ (CRL-2) and 293T (CRL-3216) cell lines were purchased from ATCC which were authenticated by ATCC. ARKL1 and RNF111 CRISPR-Cas9 KO U2OS cell lines were generated in this study.
Authentication	U2OS, Hela, BJ and 293T cell lines purchased from ATCC were authenticated by ATCC. ARKL1 and RNF111 CRISPR-Cas9 KO U2OS single clone cell lines were generated and identified by western blot. Cell lines were not further authenticated during the course of this study.
Mycoplasma contamination	Cell lines were all tested negative for mycoplasma using PCR based mycoplasma detection method.
Commonly misidentified lines (See ICLAC register)	No commonly misidentified cell lines were used in this study.
